# Eliosin-an alternative product from the Hm*PKD1* locus is a component of endoplasmic reticulum mitochondria membrane contact sites

**DOI:** 10.1371/journal.pone.0332969

**Published:** 2025-10-30

**Authors:** Almira Kurbegovic, Virginie Lazar, Wei-Min Xu, Maya Seshan, John S. Underwood, Radmila Micanovic, Jeremy M. Pomerantz, Rajneesh Srivastava, Sarath Janga, Emma H. Doud, Sherry Clendenon, Marie Trudel, Angela Wandinger-Ness, Robert L. Bacallao

**Affiliations:** 1 Molecular Genetics and Development, Institut de recherches cliniques de Montréal, Montréal, Québec, Canada; 2 Division of Nephrology, Indiana University School of Medicine, Indianapolis, Indiana, United States of America; 3 School of Informatics and Computing, Indiana University-Purdue University, Indianapolis, Indiana, United States of America; 4 McGowan Center for Regenerative Medicine, Department of Surgery, University of Pittsburgh, Pittsburgh, Pennsylvania, United States of America; 5 Biochemistry and Molecular Biology, Indiana University School of Medicine, Indianapolis, Indiana, United States of America; 6 Center for Proteome Analysis, Indiana University School of Medicine, Indianapolis, Indiana, United States of America; 7 Department of Medicine, Department of Biochemistry and Molecular Medicine, Faculty of Medicine, Université de Montréal, Montréal, Québec, Canada,; 8 Department of Pathology, University of New Mexico School of Medicine, Albuquerque, New Mexico, United States of America; University of Tartu, ESTONIA

## Abstract

The human *PKD1* gene locus region is the site that when mutated, causes 87% of the cases of human autosomal dominant polycystic kidney disease (ADPKD). This gene generates a full-length 14 kb message and encodes polycystin-1 (PC1). Informatic analysis of the *PKD1* locus reveals 38 additional transcripts in the database, the most abundant cDNA is TESTI2047494 (GenBank ACC. No. DB056008) that maps to the 3’ region with active and open chromatin. This *PKD1* locus region in human adult kidney cDNA probed by several sets of primers and sequencing produces an alternative transcript with a transcriptional start site in intron 40 that undergoes exon 42 skipping but aligns with exon 43−46 conventional splicing of the H*mPKD1* gene. To assess the broader significance of this transcript, transcriptional characterization uncovered a highly similar murine renal alternative transcript suggesting a conserved functional role. The human alternative cDNA was analyzed for protein expression and only one of three reading frames led to a 47 kDa protein that is given the name Eliosin. Eliosin protein initiates from a non-canonical translation start site Leucine in exon 41 that generates 5 unique amino-terminal amino acids in a different frame from *PKD1*. In 2D-gel analysis, Eliosin protein detected by anti-C terminal PC1 antibodies has a pI of 9.0 and the relative molecular weight was confirmed. Eliosin co-localizes with mitofusin-1, IP3R and dynamin related protein-1 (DRP1), proteins associated with ER mitochondria membrane contact sites (ERMCS). Eliosin observed in cotransfection studies with DRP1 support sequestration and/or competition mechanism at the ERMCS from classical interaction. Strikingly, exogenous Eliosin in immortalized ADPKD renal epithelial cells converts fragmented mitochondria populations to a filamentous shape. Our studies highlight the genomic complexity of the locus, a newly identified transcript and ERMCS protein, Eliosin with a role in mitochondria dynamics and potential impact in ADPKD progression.

## Introduction

The human *PKD1* gene locus is responsible for most cases of autosomal dominant polycystic kidney disease (ADPKD). ADPKD is a multisystemic disorder characterized by numerous developments and progressive enlargement of renal cysts and many extrarenal manifestations. This disorder frequently leads to end-stage renal disease in late middle age in the USA and worldwide. However, ADPKD displays large variable renal and extrarenal phenotypic expressivity that has not yet been explained.

The *PKD1* gene is located on chromosome 16p13.3, spans at least 52kb consisting of 45 intron-exon boundaries and expresses a 14-kb full length mRNA. The chromosome 16p13.1 contains *PKD1* N-terminal duplication reiterated six times and are considered pseudogenes. These pseudogenes initiated from 5′ of exon 1 and terminate in exon 31 at nucleotide 10,307 of the mRNA [1], pkdb.mayo.edu). Some of these pseudogenes contain a promoter that can generate a mRNA highly homologous (>97%) to the genuine *PKD1* gene. Genomic loci of human *PKD1* (Hm*PKD1*) is annotated with 40 transcripts as indicated in the Ensembl database (GRCh38.p12)(https://www.ncbi.nlm.nih.gov/datasets/genome/GCF_000001405.38/) [2]. Several expressed sequence tags (ESTs) have been mapped to the Hm*PKD1* locus from a genome-wide cDNA library and many of them are full-length cDNA arising from the Hm*PKD1* gene [3,4]. In addition, studies of the human *PKD1* gene revealed naturally occurring isoforms from alternative splicing, independent of differential splicing mutations considered in pathogenic variants [5]. Previous studies have reported that a human *PKD1* cDNA pool can include an additional exon in intron 16 and produced a truncated isoform [6] and another, with extra sequences in exon 17 [7]. The human *PKD1* gene also contains two long polypyrimidine tracts in introns 21 and 22 that favors aberrant differential splicing leading to premature termination and truncated *PKD1* transcripts [8]. Further, it appears that the *PKD1* gene generates 2 isoforms due to alternate splice site at the junction of intron 31 and exon 32, differing by 3 nucleotides downstream [7,9]. Sequences in the *PKD1* locus include several binding sites for microRNA miR17-5, miR20, 93,106, 519, 200, 429. Interestingly, the Hm*PKD1* locus also houses the microRNA gene miR-1225 within intron 45 [10].

The Hm*PKD1* gene is largely conserved in other species. In the mouse, the *Pkd1* gene is a 14kb gene [11,12] and does not contain gene duplication or pseudogenes. The mouse *Pkd1* gene has an identical intron/exon structure to human *PKD1* with ~80% nucleotide homology in exons and high homology in the 3’ *PKD1* intergenic region to *Tsc2* [12]. In contrast, no alternative splice forms have been reported as detected for human exons 16, 17 and 24 or in the intron21-exon22 region, consistent with the absence of polypyrimidine tracts in the mouse *Pkd1* locus. To date, the mouse *Pkd1* gene undergoes alternative splicing forms between exons 12–13 leading to different products that could be secreted [12]. Similarly to the human *PKD1* locus, the mouse locus expresses microRNA (miR) 1225 and conserves most miR binding sites.

Hm*PKD1*/m*Pkd1* encodes polycystin1 (PC1), a large transmembrane protein of ~4302aa. It has been localized to several subcellular compartments like the primary cilium, focal adhesions, the desmosome, multivesicular bodies, ER and mitochondria that support distinct cellular roles. PC1 has properties of a G−protein−coupled receptor (GPCR) [13] and an autoproteolysis-inducing domain (GAIN) [14,15] that is essential in PC1 maturation process. Mutations in the PC1 GAIN domain prevent cleavage and are associated with ADPKD phenotypes in human and in mouse [13,16]. PC1 may also generate several additional cleavage products such as a 200 aa C-terminal fragment produced by regulated intramembrane proteolysis that was shown to inhibit beta-catenin [17]. Other shorter C-terminal PC1 fragments were reported localized in the mitochondria matrix and nucleus [18,19]. The manifold shorter forms of PC1 speak to a complicated biogenesis that remains incompletely understood and/or possibly, to additional transcriptional products of the *PKD1* locus that have essentially not been investigated.

Fully understanding the genetics of *PKD1* locus is of key importance toward identification of genes embedded in the locus and could have an impact on the role of gene variations and mutations in ADPKD clinical presentation. Our study focuses on an element of Hm*PKD1*’s genomic structure. Herein, we show evidence of a *PKD1* endogenous alternative transcript produced at a start site from intron 40 that is ubiquitously expressed and abundant across multiple human tissues and validated by expression studies in kidneys. A similar homolog of this alternative transcript was also detected in mouse, supporting functional significance. Protein analysis of this alternative transcript from one open reading-frame is of expected size and PI. This protein that we named Eliosin shows localization, by chimeric constructs, to mitochondrial-ER contact sites. Most importantly, exogenous expression of Eliosin can correct the human cyst derived kidney cell’s phenotype of fragmented mitochondria.

## Materials and methods

### Bioinformatic analysis of PKD1 locus

Bioinformatic analysis of the transcriptome profile of ~53 human normal body sites was downloaded from GTEx [20–22] and expression profile of all 40 transcripts of *PKD1* were extracted. We calculated the median expression levels in Transcripts Per Million (TPM) for each body site and created a median expression matrix for *PKD1* transcripts. Resulting expression matrix was normalized by column maximum and hierarchically clustered using Euclidean distance with complete linkage using Morpheus software (Broad Institute) [23].

### Plasmids and constructs

The TESTI2047494 cDNA was supplied by Dr. Sugano (Waseda University, Japan).The clone was supplied in the pME18S-FL3 mammalian expression vector [24,25]. This clone was first identified as part of a genome-wide cDNA library construction project using validated 5’ human 5’ mRNA cap sequences [24].

The full length alternative human *PKD1* was cloned in a two-step process since the TESTI2047494 cDNA did not contain the full length 3’UTR, in the pME18S-FL3 vector. To clone the full length alternative *PKD1* sequence a PCR based strategy was employed. All primer pairs were designed using DNASTAR (Milwaukee, WI). First, the 5’ region of alternative *PKD1* cDNA was amplified using this plasmid as a template: forward PKD 1.3 and reverse PKD 4.6 primers were used for PCR amplification with GC 2 polymerase from TaKaRa Bio (Madison, WI). The thermocycler program consisted of two initial hold temperatures denaturation (98° C) for 5 minutes and 72° C for 5 minutes (for manual hot start) followed by

35 cycles as described in Table 1 and a final extension of 7 minutes. Second, the 3’-UTR of alternative *PKD1* cDNA produced from human kidney tissue mRNA (Invitrogen) with PKD 3.6 primer and then amplified using forward PKD 4.5, reverse PKD 3.4 primers with Superscript IV enzyme (Invitrogen, Thermo-Fisher Scientific Waltham, MA). Subsequently the 5’ and 3’ overlapping amplified fragments were purified using the New England Biolabs PCR purification kit (Ipswich, MA) and were joined by PCR amplification using forward Test F2 and reverse primer PKD 4.0. The anneal/extend reaction conditions were the same as above PCR amplification except for longer initial denaturation of 10 minutes and extension of 15 minutes and the PCR primers were added at the end of this extension time.

**Table 1:** PCR primer and cycling conditions employed to identify transcripts in human and mouse.

Human *PKD1* Primer	Primers position*	Primer specific sequences	Cycling Conditions (35 cycles) Figs 2 and 4
**PKD 1.5/PKD 1.6**	end ex41/ ex45	5’-TGCACAACTGGCTGGACAAC/5’-TCCACCATCTCGTAGTCCTG	98°C 30s., 67°C 30s.,72°C 2 min
**PKD 7.0/PKD 1.6**	ex41–**43**^**jct**^/ ex45	5’-AACTGGCTGGACAACAG**GTG**/5’-TCCACCATCTCGTAGTCCTG	98°C 30s., 67°C 30s.,72°C 2 min
**PKD 7.1/PKD 1.6**	ex41–**43**^**jct**^/ ex45	5’-CTGGCTGGACAACAG**GTGTG**/5’-TCCACCATCTCGTAGTCCTG	98°C 30s., 67°C 30s.,72°C 2 min
**PKD 7.2/PKD 1.6**	ex41–**43**^**jct**^/ ex45	5’-CTGGACAACAG**GTGTGCCTG**/5’-TCCACCATCTCGTAGTCCTG	98°C 30s., 67°C 30s.,72°C 2 min
**PKD 15/PKD 1.6**	early ex41/ ex45	5’-TCCTGTGCCGTGTATGACAG/5’-TCCACCATCTCGTAGTCCTG	98°C 30s., 67°C30s., 72°C 2 min
**PKD 1.3/PKD 4.6**	end in40/ mid 3’UTR	5’-ACGCCACCCCTCTCCGGCAG/5’- CTGACACGAGACACACAGTG	98°C 30s., 65°C 30s.,72°C 3 min
**PKD 4.5/PKD 3.4**	mid 3’UTR/ end 3’UTR	5’-ATTACCTCTCCAGTTCCTAC/5’-CAGTCAGACAGCTCTTTTATTG	98°C 30s., 65°C 30s.,72°C 3 min
**Test F2/PKD 4.0**	ex41/ late 3’UTR	5’-CAGCGGGGGCTACGTGCAG/5’-TGACTTTGTCTGCTTGGTGC	98°C 30s., 65°C 30s.,72°C 3 min
**Test F6 (7.1)**	ex41–**43**^**jct**^	5’-CTGGCTGGACAACAG**GTGTG**	98°C 30s., 69°C 30s.,72°C 3 min
**Test F7**	ex41–**43**^**jct**^	5’-GCTGGACAACAG**GTGTG**TGC	98°C 30s., 69°C 30s.,72°C 3 min
**Test R5**	His tag	5’-CTAGTGGTGATGGTGATGATG AGTGCTGCTGGGGTGGACCTC	98°C 30s., 69°C 30s.,72°C 3 min
**PKD 1.8**	late ex45	5’-CTGCGCAGGAAC	RT primer
**PKD 3.6**	end 3’UTR	5’-GCAGTCAGACAG	RT primer
**Mouse *Pkd1* Primer**	Primers position*	**Primer specific sequences**	**Cycling Conditions ** **Fig 3**
**11.06/11.07**	early ex41/ early ex45	5’-GCTACTGTGCAGTGTATGACA/5’- CCAGGGCACAGCACCAGGAA	50°C 2 min; 95°C 2 min; 40X (95°C 15s, 60°C 1 min)
**11.06/97.26**	early ex41/ mid ex43	5’-GCTACTGTGCAGTGTATGACA/5’- CTGTAGCTGCTGTCAGTATC	left: 95°C 5 min; 35X (95°C 50s, 60°C 50s, 72°C 1 min); 72°C 7 min right: 95°C 5 min; 40X (95°C 50s, 60°C 50s, 72°C 1 min); 72°C 7 min
**23.01/11.07**	ex41–**43**^**jct**^/ early ex45	5’-GACAGCAG**GTGTGCTTGC**/5’- CCAGGGCACAGCACCAGGAA	95°C 5 min; 33X (94°C 50s, 60°C 50s, 72°C 50s); 72°C 10 min
**23.02/97.26**	ex41–**43**^**jct**^/ mid ex43	5’-CTGGCTTGACAGCAG**GTGTG**/5’- CTGTAGCTGCTGTCAGTATC	95°C 5 min; 35X (95°C 50s, 60°C 50s, 72°C 1 min); 72°C 7 min
**11.07**	early ex45	5’-CCAGGGCACAGCACCAGGAA	RT primer

*Early: 5’ region of exon; mid: middle of exon; late: towards the end of exon; end: end of exon; jct: junction.

**Fig 1:**
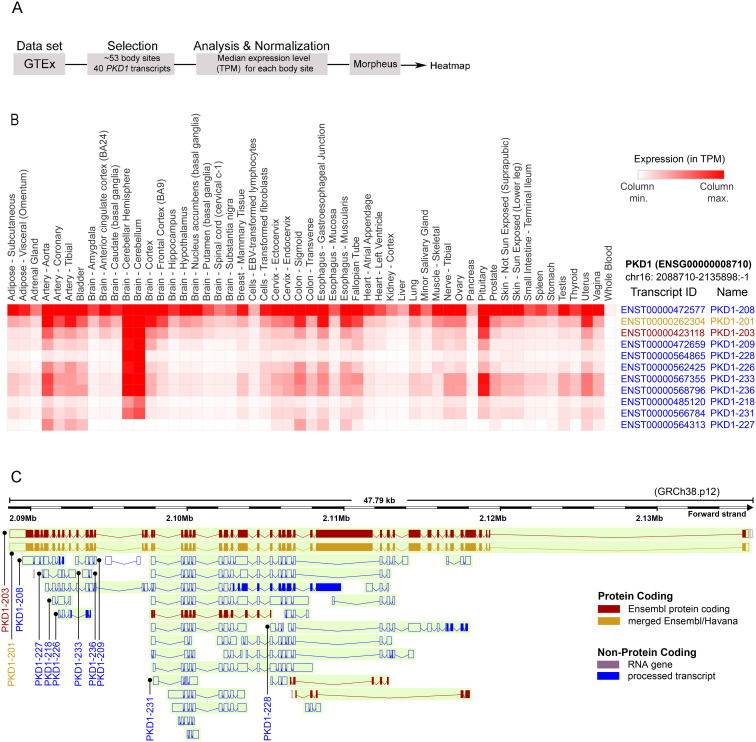
Organization of the *PKD1* locus and transcripts expression profile from GTEx. A. Workflow of analysis of public databases to generate a heat map of *PKD1* transcripts. From GTEx 40 *PKD1* transcripts were identified from 53 body sites. The median expression levels for each tissue site were evaluated and normalized. Heatmaps for transcript expression was generated via Morpheus software package (https://software.broadinstitute.org/morpheus). B. The median expression levels (in TPM) of highly expressed transcripts (normalized by column maximum) were represented in heatmap of Ensembl Transcript (ENST). *PKD1*-208 stands out as the most abundant and highly ubiquitous transcript. Full-length *PKD1* transcripts are highlighted in yellow and red. Heat map shows the color scale relative to transcript number. C. Genomic track of human *PKD1* obtained from Ensembl (GRCh38.p12), representing major transcripts. Transcripts in red, are transcripts predicted to be protein coding. *PKD1*-208 is located on the 3’ end of the Hm*PKD1* locus and is predicted to be a processed transcript.

The alternative *PKD1* full length cDNA was cloned in the backbone plasmid mCherry2-C1 (Addgene plasmid # 54563) by using EcoRI and BamHI restriction enzyme introduced into the PCR primers. To determine the protein initiation site, plasmid constructs were produced in the backbone of pENTR/pSM2(CMV/TO) (w55-1) (Addgene plasmid # 17388). The IRES GFP sequences were generated following PCR amplification of the plasmid pcDNA3.1(+) IRES GFP (Addgene plasmid # 51406). Additional restriction enzymes were incorporated in primers for cloning. The bGH polyadenylation signal was amplified by PCR from plasmid pENTR.CMV (Addgene plasmid # 32688) and cloned into backbone plasmid (pENTR CMV-TO-IRES GFP) along with a tetracycline response element (TO), an internal ribosome entry site (IRES) and a green fluorescent protein (GFP) gene sequence. Two constructions were created to determine the potential initiation site for alternative *PKD1*. Forward PCR primers Test F6 and Test F7 were designed to amplify nucleotide region of alternative *PKD1* including and excluding Leucine residue (CTG, 111 bp downstream from the start of exon 41) with the reverse primer Test R5 containing His tag sequences. The two PCR products were subcloned into pENTR CMV-TO-IRES GFP using restriction enzymes XhoI and EcoRI and final clones sequenced. DNA sequencing of plasmid constructs and recombinant clones as well as PCR amplification products were verified using DNA sequencing of ACTG services (Wheeling, IL) and contigs assembled using DNASTAR Lasergene software (Madison,WI) or IRCM internal service.

### Human and mouse endogenous full length alternative PKD1 transcript

Total RNA from normal human adult kidney tissue was purchased (Invitrogen, Waltham, MA). Briefly 2 μg of total RNA was reverse transcribed using superscript IV (Invitrogen, Waltham, MA) at 42^o^ C using *PKD1*.8 or PKD3.6 primers for one hour and subsequently incubated in 98° C for 10 minutes. The newly synthesized cDNA was subjected to hot-start PCR using Advantage GC 2 polymerase mix according to manufacturer’s recommendations (Takara Bio USA Inc. San Jose, CA) with forward and reverse primers as indicated in Table 1. The thermocycler was programmed for 2 hold temperatures, 5 minutes 98^o^ C the initial denaturation, and 10 minutes at 72^o^ C followed by 35 cycles of denaturing, annealing, and extension and final 72^o^ C extension for 7 minutes (Table 1). The purified PCR product was sequenced.

Transgenic mouse of *Pkd1*^WT^ (originally *Pkd1*_TAG_ 26 line) (27), and ^SB^*Pkd1* (originally ^SB^*Pkd1*_TAG_^line 41)^ [26] both bred to the wild-type C57BL/6J genetic background for several generations. *Pkd1*^WT^ and ^SB^*Pkd1* overexpress full-length mouse *Pkd1* gene systemically or preferentially in renal epithelial cells, respectively. All animal experimental protocols were approved by the Animal Care Committee of the Institut de Recherches Cliniques de Montréal and conducted according to the guidelines of the Canadian Council on Animal Care. RNA renal extracts preparations were produced as previously reported [27]. DNAse-digested RNA (0.25 μg or 0.4 μg) were reversed transcribed with M-MLV (ThermoFisher Scientific) and random [d(N)6] hexamers (0.025 μg/μl or 0.1 μg/μl) or 11−7 primer (0.5μM) for 45 min at 37°C in a final total volume of 20ul. The conditions and primers for PCR and quantitative PCR reactions for analysis of murine kidney cDNAs are summarized in Table 1.

### Cell culture and transfection

NIH 3T3 was transfected with pME18S-FL3-TESTI2047494 or pGreen using TurboFectin 8.0 (OriGene, Rockville, MD). Protein lysates were made by solubilizing cells 48 hours post transfection with 140 mM NaCl, 25 mM Tris-Cl, pH 7.4, 1 mM EDTA, 0.5 mM EGTA, 1% Triton X-100, 0.1% SDS, 1 mM phenylmethylsulfonyl fluoride (PMSF), 10 µM chymostatin, leupeptin, aprotinin and pepstatin. DNA within the lysate was sheared by 10 second bursts for 1 minute with a probe sonicator at 4^o^ C.

Human embryonic kidney 293 (HEK293) (American Tissue Type Collection) or human WT 9−7 ADPKD cells [28] were cultured in DMEM media (ThermoFisher Scientific, Waltham, MA) with 10% fetal bovine serum and in a 37°C 5% CO_2_ incubator was seeded at 50% confluence a day before transfection. Cells were co-transfected as described for NIH 3T3 with mCherry2 C1 containing the alternative *PKD1* cDNA and SNAP-Drp1 plasmids. SNAP-Drp1 mitochondria localization/fission marker and SNAP substrate were purchased (Addgene plasmid # 141159; New England Biolabs, Ipswich, MA) [29]. Fluorescence conjugated secondary antibodies were purchased from Jackson Immuno research (West Grove, PA). The microscopy images were taken after 48 hours of transfection with Olympus microscope (Indiana University O’Brien Center). HEK 293 cells were used to transfect plasmids pENTR CMV-TO-IRES GFP alternative PKD (- CTG and or +CTG) leucine residue and protein lysates collected after 72 hours for expression analysis. Cell line confluence prior to transfection experiments was visually assessed by transmission light microscopy and transfection success was determined by fluorescence light microscopy whenever a plasmid expressing a fluorescent protein was used in the experiments.

HK-2 cells were transfected with 1 µg siRNA with MACSfectin (Miltenyi Biotec, Gaithersburg, MD) to knockdown polycystin-1 expression. The following antisense constructs were purchased (Milipore Sigma, Burlington, MA): SASI-Hs02–00302280 (nucleotide start site 505 in *PKD1* gene), SASI-Hs02–00302278 (nucleotide start site 12243 in the *PKD1* gene), SASI-Hs01–00136684 (nucleotide start site 4548 in the *PKD1* gene), SAAI-Hs01–00136685 (nucleotide start site 3498 in the *PKD1* gene) (Milipore-Sigma, Burlington, MA). In all experiments, an experimental group of cells were treated with Mission siRNA Universal negative control (Millipore-Sigma, Burlington, MA). Forty-eight hours after transfection, cell lysates were obtained by solubilizing proteins in RIPA buffer. All lysates were sonicated to shear DNA in the lysate. Immunoblots were performed as previously described [30].

### Analysis of TESTI2047494 cDNA open reading frame

The pME18S-FL3-TESTI2047494 was digested with EcoRI and HindIII and the purified fragment was cloned into cTAP A, B and C. HEK293 cells were transfected with TESTI2047494 or cTAP A, B or C using Lipofectamine 2000. Two days after transfection cells were lysed with 50 mM Tris-HCl, pH 8.0, 150 mM NaCl, 1% Triton X-100, 0.1% sodium dodecyl sulfate, 0.5% sodium deoxycholate, 1mM EDTA, 1 mM phenylmethylsulfonyl fluoride (PMSF) and 1 µM chymostatin, leupeptin, aprotinin and pepstatin. Lysates were sonicated to shear the DNA. Protein concentration of the lysates was determined using the BCA protein assay (ThermoFisher Scientific, Waltham, MA) and bovine serum albumin as a reference control. Equivalent amounts of total protein (20 µg) were loaded on a 10% Tris-glycine polyacrylamide gel (Thermo Fisher Scientific, Waltham, MA). Immune blots were performed as previously described and blots were probed with anti-calmodulin antibody (RRID: AB_11212268) [30–32].

### Protein characterization

For Eliosin and Pc1 immunoblotting, proteins were transferred onto nitrocellulose membrane using 25 mM Tris, 192 mM glycine, pH 8.3, 20% MeOH, 0.1% sodium dodecyl sulfate (BioRad,Hercules, CA). The blots were blocked (2% newborn calf serum, 10 mM Tris-Cl, pH8.0, 150 mM NaCl), probed with NM005 an anti-human C-terminal PC1 antisera (34) and washed three times (10 mM Tris-Cl, pH. 8.0,150 mM NaCl, 1% Triton-X 100), then with (10 mM Tris-Cl, pH 8.0, 500 mM NaCl) and then with (50 mM Tris-Cl, pH 8.0). Blots were developed with Super Signal (ThermoFisher Scientific, Waltham, MA). All blots were stripped with Restore Western Blot Stripping Buffer (ThermoFisher Scientific, Waltham, MA) and then probed with anti-actin antibody to evaluate equivalent loading. For the 2D gel electrophoresis, proteins were solubilized with 8 M urea, 2% 3-[(3-cholamidoproply) dimethylammonio]-1-propanesulfonate (CHAPS), 50 mM DTT, 0.2% (w/v) Bio-Lyte 3/10 ampholytes and trace Bromophenol Blue (BioRad, Hercules, CA). Protein concentration was determined in each extract using the Cytiva 2D Quant kit (Sigma-Aldrich, St. Louis, MO). 50 µg of total protein was loaded onto pH 3–10 ReadyStrip IPG 11 cm strips. The strips were rehydrated for 16 hours at 10^o^ C. The proteins were then focused to a total of 75,000 Volt-hours at 20^o^ C. After isoelectric focusing, the strips were equilibrated in 6 M urea, 2% sodium dodecyl sulfate, 0.375 M Tris-Cl, pH 8.8, 20% glycerol and Bromophenol Blue. Second dimension runs were performed on 4–12 gradient gel (BioRad, Hercules, CA). After 2D gel electrophoresis, immune blots was performed by transferring proteins onto nitrocellulose membrane and probed with Polycystin1 C-terminal antisera (NM005) [33].

For studies where we analyzed the protein initiation site for Eliosin the cDNA of the alternative transcript was cloned in pENTR CMV-TO-IRES GFP plasmid. Protein expression was analyzed by immunoblotting probed with anti-His antibodies (Genescript, Nanjing, China; RRID AB_3676335.

### Proteomic, mass spectrometry LC-MS/MS and data analysis

For proteomic analysis, transfected His-tagged Eliosin expressed by HEK293 cells was eluted from a nickel column, the sample was diluted with an equal volume 8 M urea in 100 mM Tris HCl, pH8.5, reduced with 5 mM tris (2-carboxyethyl) phosphine hydrochloride (TCEP, Sigma-Aldrich, St. Louis, MO) for 30 minutes at room temperature and alkylated with 10 mM chloroacetamide (CAA, Sigma Aldrich, St. Louis, MO) for 30 min at room temperature in the dark. The reduced and alkylated protein sample was then split into two tubes for separate trypsin and chymotrypsin digestion. One sample was diluted to 2 M Urea with 50 mM Tris HCl, pH 8.5 and digested overnight at 35º C with 0.25 µg Trypsin/Lys-C (Promega Corporation, Madison, WI). The other sample was diluted with 10 mM CaCl_2_, 50 mM Tris pH 8 to 0.5 M final urea concentration and digested overnight at 35º C with 1 µg chymotrypsin (Millipore Sigma, St. Louis, MO). Digestions were quenched with trifluoracetic acid (TFA, 0.5% v/v) and desalted on Waters Sep-Pak cartridges (Waters, Milford, MA) with a wash of 1 mL 0.1% trifluoracetic acid followed by elution in 50% and 70% acetonitrile containing 0.1% formic acid in the same collection tube. The eluates were dried and stored at −20º C.

Eluted protein digests were resuspended in solvent A (0.1% formic acid) and fully injected using an EasyNano1200 LC (Thermo Fisher Scientific, Waltham, MA) onto an EasySpray column and separated over a 120-minute method (4–28% solvent B over 110 minutes, increasing to 80% solvent B (80% acetonitrile, 0.1% formic acid) over 5 min, hold at 80% solvent B for 1 min and decreasing to 4% solvent B over 4 min. FAIMS pro was utilized with an Exploris 480 mass spectrometer (Thermo Fisher Scientific, Waltham, MA). Three high field asymmetric ion mobility spectroscopy (FAIMS) CVs were used with 1.3 sec cycle times (−40, −55, −70 V). MS 1 settings: scan range 375–1500 m/z, RF lens 50%, standard AGC target, auto max inject time, minimum intensity 5e3, charge states 2–7, shared dynamic exclusion of 30 sec excluding isotopes. MS2 scan settings: isolation window 1.6 m/z, isolation offset off, fixed collision energy of HCD 30%, orbitrap resolution of 15,000, standard AGC target auto max IT. Raw data files were analyzed using PEAKS Studio Xpro 10.5 build 20211221 (Bioinformatics solutions). *De novo* sequencing was performed with 10 ppm precursor mass error tolerance, 0.2 Da fragment ion mass tolerance, enzyme specified by each sample (trypsin with D, P and chymotrypsin, respectively). Database searches were done using a Uniprot *Homo sapiens* database with the addition of *PKD1* 420 AA sequence and common contaminants (20479 sequences in total), semi specific digestion, maximum of 3 missed cleavages, variable modifications of N-terminal acetylation, oxidation on M, phosphorylation on S, T, or Y, and carbamidomethylation, maximum of 3 missed cleavages and maximum of 3 variable mods per peptide. Peaks PTM was then used to search for 314 additional post-translational modifications. Data was filtered for 1% FDR at the peptide level and de novo scores of >80.

### Mitochondria circularity

Mitochondria were labeled with MitoTracker Green (ThermoFisher Scientific, Waltham, MA) and images were collected as described below. Mitochondria circularity was determined using the circularity plug-in available in Fiji open-source software running on an HP laptop [34,35]. Circularity data was analyzed by one-way ANOVA with significance determined at p < 0.05.

### Image acquisition

Images were collected with an Olympus Flowview FV1000 confocal module mounted on an Olympus iX 81 inverted microscope (Olympus Life Sciences, Center Valley, PA). Excitation light was provided by a Spectraphysics DeepSee tunable titanium-sapphire laser at 800 nM femtosecond pulsed laser (Spectra-Physics, Santa Clara, CA) or in single photon excitation mode with excitation at 405 nm, 488 nm, 514 nm. All images were collected with a 60X water immersion lens (Olympus Life Sciences, Center Valley, PA). All images collected in single photon mode, were collected sequentially to avoid image crosstalk. Data collection from all samples was performed under identical intensity, black level and line scan settings. Image processing was performed using FIJI open-source software running on an HP laptop. In experiments where co-localization was quantified a pixel-wise method was used to determine co-localization using Pearson’s correlation coefficient.

## Results

### Analysis of human *PKD1* locus

Human *PKD1* (ENSG00000008710) is located on chr16: 2,088,710–2,135,898. This locus encodes 40 transcripts including six protein coding transcripts as annotated in Ensembl database (Figs 1B and S1). We extracted the median expression profile of *PKD1* transcripts from GTEx (See methods, Fig 1A). The expression matrix was normalized by column maximum and represented as hierarchically clustered heatmap (Fig 1B). We observed that transcript *PKD1*–208 (ENST00000472577) was highly expressed and hierarchically out-grouped from other transcripts (Figs 1B and S1). A subset of transcripts expressed in multiple tissues with high median expression is shown in Figs 1B and S1. The transcript labeled *PKD1–208* (ENST00000472577) (Figs 1B and S1) is the most abundant and ubiquitously expressed transcript except for whole blood where the expression is much lower as compared to other tissues (S1 Fig). These transcripts were labeled into the genomic track of *PKD1* with hg38 coordinates obtained from Ensembl GRCh38.p12 (Fig 1C). We noted transcript labeled ENST00000472577 which is identified as a transcript mapped to the *PKD1* loci in the AceView database with accession number DB 056008. DB 056008 is a cDNA called TESTI2047494. This transcript maps to the *PKD1* intron 40 on its 5’ end and is predicted to have a size of approximately 1.1 kb. Due to the unique intronic start site of the transcript and its frequency of expression, we pursued an investigation into the function of this transcript.

### Evidence of an alternative human transcript within the *PKD1* locus

**Fig 2:**
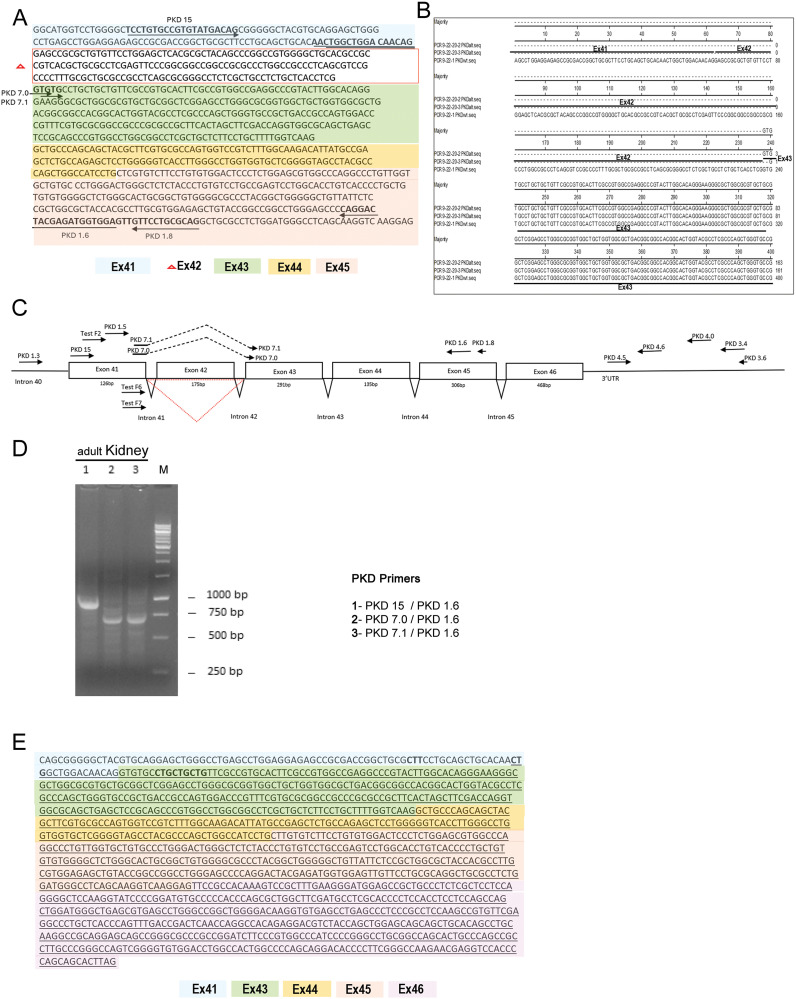
Sequence and detection of human *PKD1*-208 alternative transcript. A. Nucleotide sequence of wild type *PKD1* transcript, the red boxed nucleotides delineate exon 42 which is deleted in alternative *PKD1* transcript (*PKD1-208*). Bold sequences are primer sites. Each exon has been color coded. B. Pairwise alignment of nucleotide region for alternative *PKD1* transcript and wild type *PKD1*, showing missing exon 42 in the alternative transcript. C. Schematic diagram of intronic and exon sequences from full-length Hm*PKD1* starting from intron 40. *PKD1*-208 transcript has an exon 42 skipping (triangle below exon). PCR primers used in this study are mapped to the diagram (nucleotide sequences are found in Table 1). D. Detection of wild type and alternative *PKD1* transcript in human kidney tissue using common *PKD1* primers (Listed on the left of the agarose gel image) (lane 1) and specific primers (lane 2 and 3) for the alternative transcript. M, 1Kb DNA ladder. E. Sequence of *PKD1*-208 cDNA with same color code as in A. Underlined nucleotide sequences represent protein coding region of alternative *PKD1* transcript. Bold sequence CTG are potential Leucine translation start sites.

The DNA sequence of *PKD1*–208 within the human *PKD1* locus has several notable features (Fig 2A). The alignment of the *PKD1*–208 cDNA with the pertinent region of full-length Hm*PKD1* is shown in Fig 2B. The first 37 bases are sequences from intron 40 from the Hm*PKD1* locus. The intronic sequence is followed by sequences from exon 41 and then there is a splice from the 3’ end of exon 41 to exon 43 in the Hm*PKD1* locus. Subsequently, the sequence has the normal splices between exons 43, 44, 45 and 46 (Fig 2B) such that the derived amino acid sequence would be predicted to match the carboxyterminal amino acid sequence from polycystin-1. Further inspection of the cDNA reveals that there is no Kozak sequence and there is no canonical ATG start site [28]. A schematic diagram of the Hm*PKD1* gene region from which full-length polycystin-1 mRNA is created, is compared to *PKD1–208* (Fig 2C). To determine if the *PKD1*–208 transcript is expressed, we performed RT-PCR studies using total RNA from adult human kidneys. Since exon 42 is spliced out of *PKD1–208*, any PCR reaction using primer pairs that flank the exon 42 should result in a longer product when *PKD1* mRNA is the source of the product as compared to *PKD1–208*. Fig 2D shows that a PCR product using total kidney RNA as a template and primer pair *PKD 1*5/PKD 1.6 yields an indistinct band that runs between the expected 790 bp for the alternative transcript and 965 bp for full-length PCR fragment (Fig 2D, Lane 1), perhaps a mixture of two products or only the highly expressed alternative transcript. To ensure that we detect an alternative transcript directly, primer pairs PKD 7.0/ PKD 1.6 or PKD 7.1/ PKD 1.6 specific for the splice junction are used in the reaction, a ~ 700 bp product is observed (Fig 2D, Lanes 2 and 3). The predicted smaller size products (695 and 697 bp) due to the exclusion of exon 42 from the PCR fragment (Fig 2D) are migrating at the appropriate size. This data confirms that the alternative transcript *PKD1*–208 is expressed in the kidney. The sequence of *PKD1–208* is presented in Fig 2E showing the region of the partial exon 41 and the contiguous sequence containing exons 43–46 of Hm*PKD1*.

### Characterization of a homologous alternative *Pkd1* transcript in mouse

**Fig 3:**
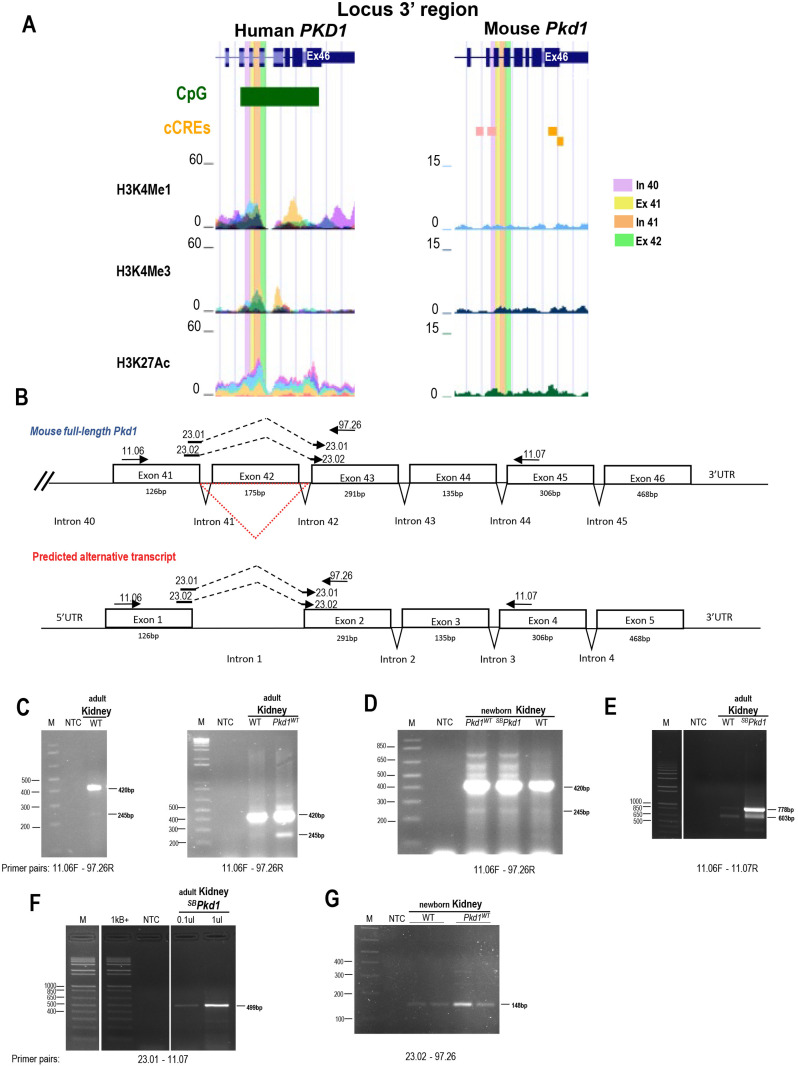
Identification of a murine alternative transcript within the *Pkd1* locus. A. Transcriptional activity of the human (left) and mouse (right) 3’ end *PKD1*/*Pkd1* locus from UCSC Browser (exons 39 to 3’UTR). This human region has high CpG density (green box). Alignment of ChIP-Seq binding pattern data of selected chromatin marks show strong chromatin activity in various human cell lines (NHEK, HUVEC, K562, NHLF, H1-hESC, GM128878) but weak in the mouse newborn kidney genome. Mouse intron 39 to exon41 contains two candidate cis-regulatory elements (CRE) marks indicative of promoter-like biochemical signature from H3K4Me3 (pink box). Mouse exon 46 and 3’end UTR contain two proximal enhancer-like signatures (orange box). B. Schematic diagram of mouse 3’ end *Pkd1* locus from intron 40 to 3’UTR with corresponding exon size length, with or without exon 42 (red triangle). PCR primers used are mapped over this genomic region. C. Detection of corresponding human alternative transcript in total adult mouse kidney cDNA of endogenous wild-type C57BL/6J (WT) yield *Pkd1* band of 420 bp and alternative transcript band of 245 bp (primers 11.06-97.26), and no band in non-template control (NTC) (Left and right panels) and transgenic kidneys *Pkd1*^*WT*^ (right panel). Marker: 1Kb Plus DNA ladder. D. Expression of alternative transcript was monitored in newborn kidneys of endogenous WT, transgenic kidneys *Pkd1*^*WT*^ and ^*SB*^*Pkd1* with same primers as in C. E. Detection of alternative transcript in total adult mouse kidney cDNA with another set of primers (11.06-11.07). Endogenous WT and transgenic kidneys ^*SB*^*Pkd1* yield 2 bands at 778 and 603 bp for *Pkd1* and alternative transcript bands, respectively. F. Detection of alternative transcript over exon 41-43 junction in adult transgenic ^*SB*^*Pkd1* produced the predicted single band of 499 bp (primers 23.01-11.07) at different intensity according to cDNA quantity. G. Detection of alternative transcript over exon 41-43 junction in two newborn WT and transgenic *Pkd1*^*WT*^ kidneys (primers 23.02-97.26) was shown by a single band of 148 bp.

We then interrogated whether the *PKD1*–208 transcript is specific to human kidneys or is a common alternative transcript in mammalian kidneys. Since the mouse *Pkd1* locus is highly homologous to the human locus, we first compared human and mouse transcriptional activation (http://genome.ucsc.edu [10,36–38]; S2A Fig) with a closer view in the region preceding exon 40 to the 3’UTR (Fig 3A). The human *PKD1* locus appears transcriptionally active with an open chromatin status in three regions: the 5’ end; the exon 16–17 consistent with the reported alternative splicing; the exon 39 to intron 40. In addition, the human exon 40–46 region is of high CpG density, generally associated with active gene expression and with a crucial role in gene regulation [39]. While the chromatin of mouse *Pkd1* locus does not appear as transcriptionally active as the human locus, the region of intron 39-exon40-intron40 contains biochemical signature of two promoter-like elements, suggesting that this region could also be active in the mouse. As a complementary approach, we then monitored for additional transcriptional activity by scanning the locus (S3 Fig). The mouse *Pkd1* locus was tested for transcribed sequences by assessing cDNA from wild type mouse kidneys C57BL6/J at P20 with different sets of primers by qPCR. The resulting mouse amplicons in the 5’ region from exon 1 to exon 40 under melting curve analysis showed a single peak and one product with no evidence of additional transcripts (Fig S3A and S3B). Similarly, only one peak was produced from amplification of exon 40–42 since the *PKD1*–208 does not encode exon 42. In contrast, double peaks from the melting curve were clearly detected from exon 41 to the 3’ end of the *Pkd1* transcript region and appearance of extra amplicons, supporting more than one transcript and possibly, a more complex transcriptional region. Fig 3B shows a schematic diagram of the corresponding mouse alternative *Pkd1* region and primers used to test for potential transcriptional activity. To examine more precisely the putative mouse alternative transcript, the cDNA from adult wild type kidneys was amplified by PCR using primers in exon 41 and exon 43, and detected a doublet, at expected size of 245 bp for alternative *PKD1*–208 transcript and of 420 bp for full-length mouse *Pkd1* (Fig 3C left). This doublet was confirmed by using the cDNA of adult kidneys from transgenic *Pkd1*^*WT*^ mouse (~15 copies) [40] (Fig 3C right). Expression analysis at an earlier timepoint on cDNA from newborn kidneys of wild type, transgenic *Pkd1*^*WT*^ and ^*SB*^*Pkd1* (~10 copies) showed also a clear band for an alternative transcript of weak intensity (Fig 3D). The size of the higher additional bands observed in newborn transgenic kidneys corresponds to that of partially spliced or unspliced *Pkd1* primary RNA transcript or pre-mRNA. The predicted sizes are 534, 617 bp and 731 bp which are observed in Fig 3D. The presence of the alternative transcript was then corroborated by PCR produced using another set of primers in exon 41 and exon 45 on wild-type C57BL6/J, ^*SB*^*Pkd1* that resulted in two amplicons of expected size (603 and 778 bp) for alternative transcript and full-length (Fig 3E). To confirm the presence of an alternative *PKD1*–208 transcript, two forward primers (23–01 and 23–02) that cover the exon 41–43 junction with intermediate and minor coverage into exon 43 were used with a reverse primer in the exon 45 or exon 43, respectively. This approach should only target the alternative transcript excluding the exon 42, upon amplification on mouse newborn wild type, transgenic *Pkd1*^*WT*^ or adult ^*SB*^*Pkd1* kidney cDNA by PCR. A unique amplicon of 499 bp was generated from the PCR intermediate junction coverage (Fig 3F) as expected and was isolated and sequenced to confirm the appropriate junction (S5A Fig). Similarly, the primer with minor junction coverage also resulted in the predicted product of 148 bp for both wild types, transgenic *Pkd1*^*WT*^ (Fig 3G). While the expression of mouse alternative transcript appears less abundant than the human alternative transcript, our data provide evidence of an active endogenous alternative transcript in both human and mouse (alignment provided in S5B Fig).

### Identification of a unique protein Eliosin encoded by the alternative human transcript

**Fig 4:**
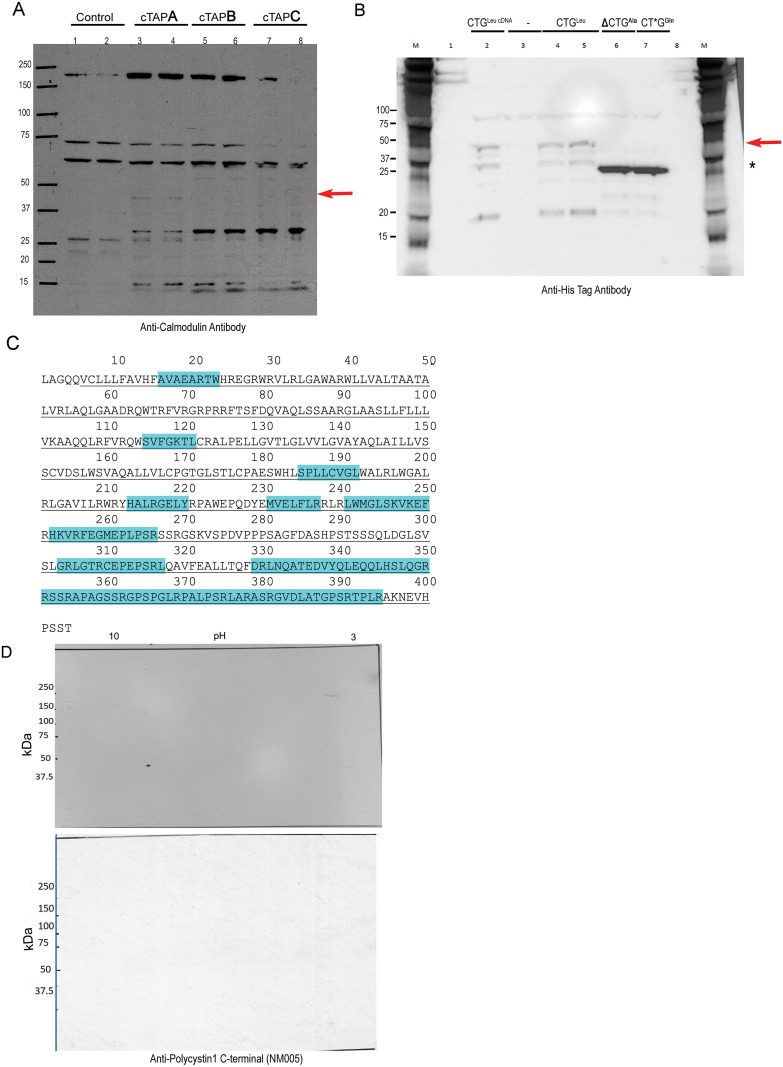
Characterization of the protein encoded by the alternative transcript: Eliosin. A. Immunoblot detection of a protein derived of the full-length alternative transcript from the cloned cDNA in three reading frames of cTAP A, B or C transfected into HEK293 cells. When in frame, expression of a chimeric protein with the calmodulin binding protein sequence at the carboxy-terminal is detected with a calmodulin specific antibody. Lanes 1, 2 mock transfected control, lanes 3, 4 transfected with cTAP A, lanes 5, 6 with cTAP B, lanes 7, 8 with cTAP C. In comparison to the mock transfected cells, a 48 kDa CBP-tagged protein is uniquely detected (red arrow) in lanes 3 and 4 from HEK293 cells transfected with cTAP A. B. Analysis of the protein initiation site was performed on the cDNA of the alternative transcript with the native 5’ UTR and ORF in pENTR CMV-TO-IRES GFP plasmid (Lane 2) and in two different clones by PCR constructs which include CTG/Leucine initiation site (Lanes 4 and 5) produced ~ 47kDa band. Lane 3 contains an empty plasmid backbone as background control. Plasmid constructs with the first three nucleotides encoding Leucine deleted, making way for Alanine to be the first potential codon (Lane 6), or mutated in the 2^nd^ base of the CTG initiation site, that should convert Leucine to a Glutamine codon (Lane 7), prevented production of the ~ 47 kDa band. The ~ 37kDa (*) band may correspond to another downstream translation start site for this alternative cDNA. Lanes 1 and 8 are empty wells. C. Amino acid sequence of isolated Eliosin with underlined sequence shared with PC1 and with highlighted amino acids representing peptide mapping coverages from proteomic studies. The first five amino terminal amino acids are uniquely expressed in Eliosin. D. 2D Gel Immunoblot analysis on NIH 3T3 cells transfected with cDNA of the full-length alternative transcript (top panel) or control pGreen (bottom panel) were probed with anti-human C-terminal PC1 (NM005) to detect Eliosin. A single spot with an approximate pI of 9.0 and a molecular weight of ~47 kDa is seen in the transfected cells in comparison to control.

Since there was no canonical ATG sequence near a Kozak sequence, studies were performed to determine the open reading frame of the human alternative transcript and the start site of the coding region. The transcript was cloned into all three reading frames of cTAP plasmid to determine an open reading frame. TAP plasmids are mammalian expression vectors with a carboxy terminal calmodulin binding protein sequence (CBP) that allows for immune detection of a protein product [31]. Despite some bands detected in the mock transfected controls, a single band (arrow) of estimated molecular weight of 48 kDa tagged-CBP protein is observed in just one reading frame (Fig 4A, arrow), indicating that the alternative transcript encodes a bona fide protein. To determine the start site of the protein, we performed site directed mutagenesis on the first leucine encoding codon. We found that deletion of the corresponding leucine codon and initiation with the following codon alanine (Lane 6) or mutation in the second nucleotide of leucine to create a glutamine codon (Lane 7) inhibited expression of the ~ 48 kDa protein normally obtained from the unmodified leucine (lanes 2, 4 and 5) (Fig 4B). The initiation start site produces 5 amino acids at the N-terminal region that are unique to this novel protein and encoded from the distal portion of *PKD1* gene exon 40. A second band at 37 kDa is observed in lanes 2 and 4 suggesting that there could be a second start site for protein translation from the cDNA. Fig 4C shows the amino acid sequence of the 48 kDa protein that we name Eliosin with the peptides identified by proteomic analysis of the isolated protein highlighted in blue. Based on the analysis of the open reading frame, 399 amino acids in the carboxy terminal region are also expressed in the carboxy terminal of Polycystin-1 (Fig 4C, underlined amino acid sequence). From this information, we ascertained that antibodies raised against unique amino acid sequences that are specific to the human Polycystin-1 protein, would bind to Eliosin particularly if the transcript is expressed in a non-primate cell line as it does not detect murine Pc1 expression. To explore the biochemical properties of Eliosin, the plasmid encoding Eliosin was transfected into NIH-3T3 cells and 2D gel electrophoresis was performed on protein lysates from Eliosin and mock transfected cells. Immune blots were performed using anti-Polycystin1 C-terminal (NM005) [33]. Fig 4D shows that a single protein spot is detected when the transcript is expressed. The relative molecular weight is 47 kDa with a pI of 9.0. The molecular weight and isoelectric point are equivalent to calculated metrics based on the amino acid sequence.

**Fig 5:**
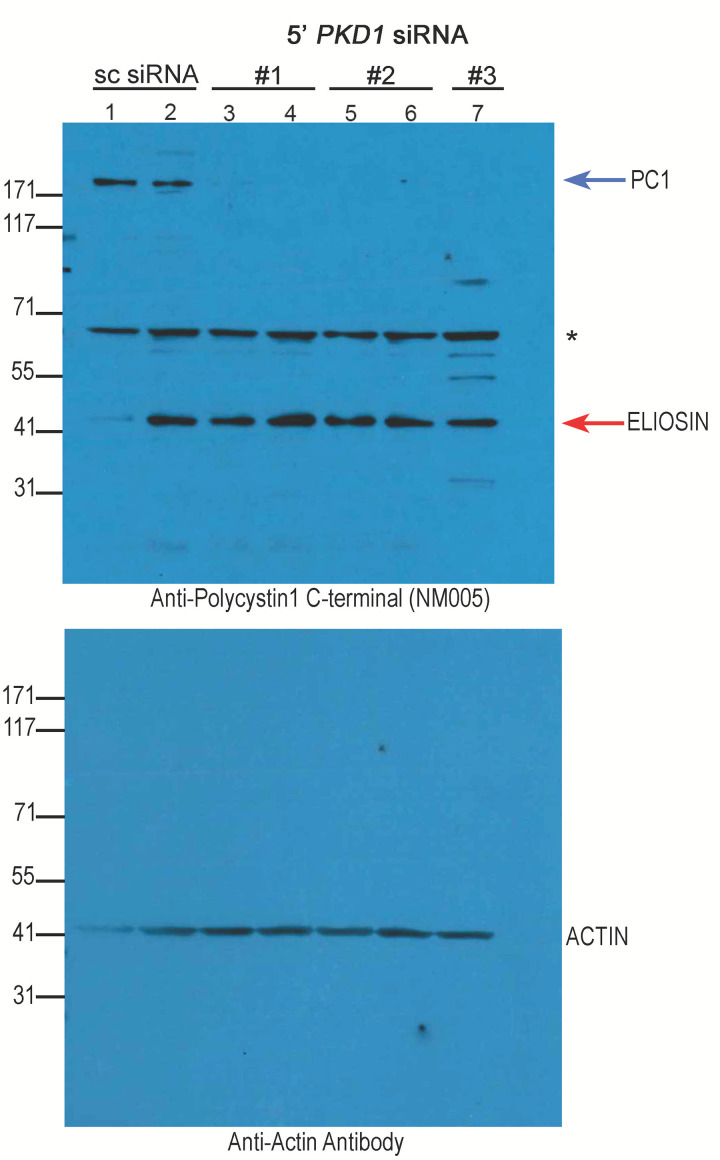
Eliosin is expressed in human cells treated with PC1 siRNA. Immunoblot of HK2 cells monitored for endogenous Eliosin expression was probed with anti-Polycystin C-terminal NM005 (Top panel). In lanes 1, 2, HK-2 cells were treated with scrambled siRNA or with 3 different siRNAs that bind to sequences located at the 5’ end of the Hm*PKD1* gene: siRNA-1, lanes 3,4; siRNA-2, lanes 5,6; siRNA-3, lane 7. A band of ~45-47 kDa corresponding to Eliosin and a high molecular weight band likely the full-length PC1 were detected in the scrambled siRNA treated cells (lanes 1, 2). The specific *PKD1* siRNAs suppressed full-length PC1 whereas the expected band for Eliosin is unaffected. The ~ 68 kDa band (*) may be an Eliosin heteromer or non-specific background band. Lower image: Same immunoblot from the upper panel probed with anti-actin.

To show definitively that Eliosin is normally produced in cells, we performed immunoblot studies on cell lysates in which full-length Polycystin1 expression was decreased by designing siRNA constructs that bind at the more 5’ regions of the Hm*PKD1* gene. This is the only approach that can be taken due to the lack of specific antibodies that can differentiate Eliosin and Polycystin1. In cells treated with the scrambled control siRNA, higher molecular weight proteins are identified in the gel using the anti-Polycystin1 C-terminal specific antibody, NM005 (Fig 5A, lanes 1 and 2). Under conditions of *PKD1* siRNA where full-length Polycystin-1 expression would be expected to be decreased (Fig 5 top panel lanes 3−7, blue arrow), Eliosin expression is still highly expressed (Fig 5, top panel lanes 3−7, red arrow) with a 48 kDa band observed. This data confirms that Eliosin is normally expressed in HK-2 cells and its expression may be misinterpreted as a lower molecular weight fragment of endogenous Polycystin-1.

### Co-localization of Eliosin with endoplasmic reticulum mitochondria contact sites

**Fig 6:**
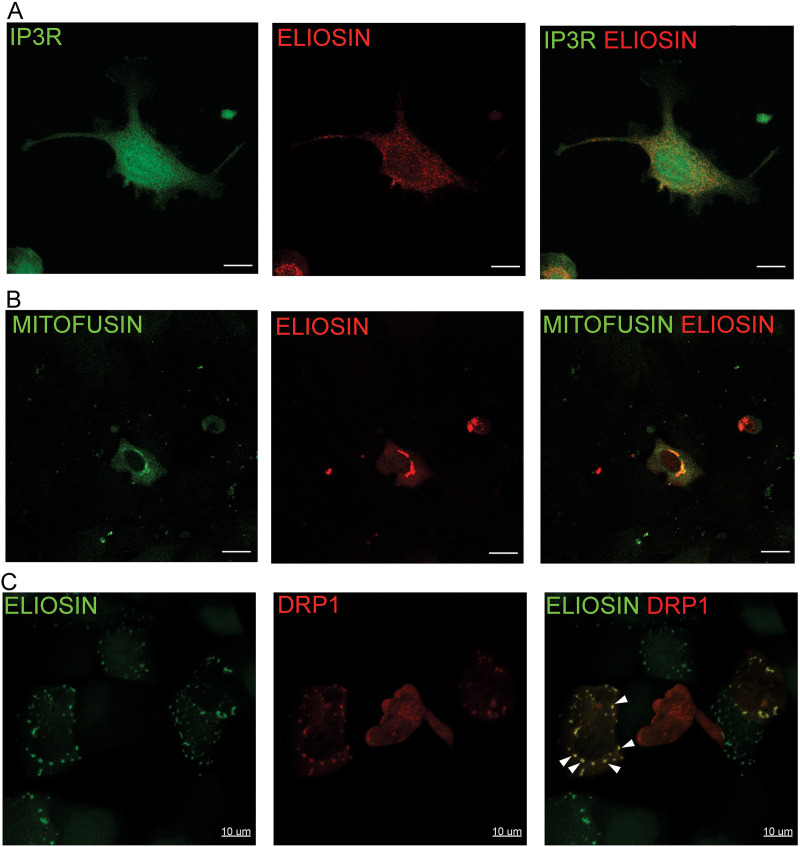
Eliosin is a component of mitochondria-ER associated membranes. A. Co-localization studies of mCherry Eliosin transfected in HEK 293 by immunofluorescence with anti-Polycystin1 C-terminus, NM005 (middle panel) and ERMCS/MAM marker IP3R (left panel). Notable co-localization between Eliosin and IP3R is observed. Bar = 10 microns. Pixel size 0.11 µm^2^, z distance = 0.35 µm per stack. The image is an extended focus image generated by projecting the maximal pixel intensity onto a single plan determined by a ray trace perpendicular to the z stack [44]. B. Immunofluorescence studies on transfected mCherry Eliosin in HEK 293 with the ERMCS/MAM marker Mitofusin 2-GFP show significant co-localization. Bar = 10 microns. Voxel size = 0.28 µm^3^. The image is an extended focus image. Z distance = 0.28 µm. C. Co-localization studies of SNAP-tagged Eliosin (green, left panel) co-transfected with mCherry dynamin related protein (DRP1) (red, middle panel) by immunofluorescence. DRP1 and Eliosin co-localize in a punctate pattern (arrowheads). Bar = 10 microns, voxel size = 0.28 µm^3^, Z = distance 0.28 µm. The image is an extended focus image.

To investigate Eliosin subcellular localization, immunofluorescence studies were first undertaken from Eliosin transfections. Our analysis yielded Eliosin localization compatible with mitochondria staining patterns, but these studies showed only ~50% co-localization with mitochondria markers. We then considered the possibility that Eliosin co-localized with the endoplasmic reticulum (ER) but co-localization studies with an ER marker showed only ~50% co-localization. These findings led us to consider the possibility that Eliosin is a component of mitochondria-ER contact sites. We find that cells transfected with Eliosin, immunostained using anti-Polycystin1 C-terminal, and co-stained for endogenous IP3R show extensive co-localization (Fig 6A). Since IP3R is a component of ER mitochondria contact sites (ERMCS/MAM) [41–43], we performed additional experiments to validate this finding.

To further investigate Eliosin subcellular localization and potential implication in the ERMCS/MAM, cells were co-transfected with Eliosin chimeric protein and a mitofusin-2 green fluorescent protein (GFP) chimeric protein and showed extensive co-localization confirming that Eliosin is localized to mitochondria-ER contact sites (Fig 6B). Co-transfection of Eliosin SNAP tag labeled green and dynamin related protein-1, DRP1 mCherry show their colocalization as illustrated in Fig 6C (arrowheads). The colocalization of Eliosin with DRP1 exhibit similar punctate and colocalizing pattern, suggesting possible interaction of Eliosin at the ERMCS/MAM. In other experiments however, DRP1 was mainly localized to the cytosol (S4 Fig), suggesting that DRP1 recruitment at the ERMCS/MAM is hindered. This data supports the conclusion that Eliosin is a component of mitochondria-ER contact sites, but the redistribution of DRP1 suggests that Eliosin’s function is to displace DRP1 from the contact sites. Since DRP1’s catalyzes mitochondria fission, we next evaluated the effect of Eliosin expression on mitochondria morphology.

### Eliosin expression improves the fragmented mitochondria defect in ADPKD renal cells

**Fig 7:**
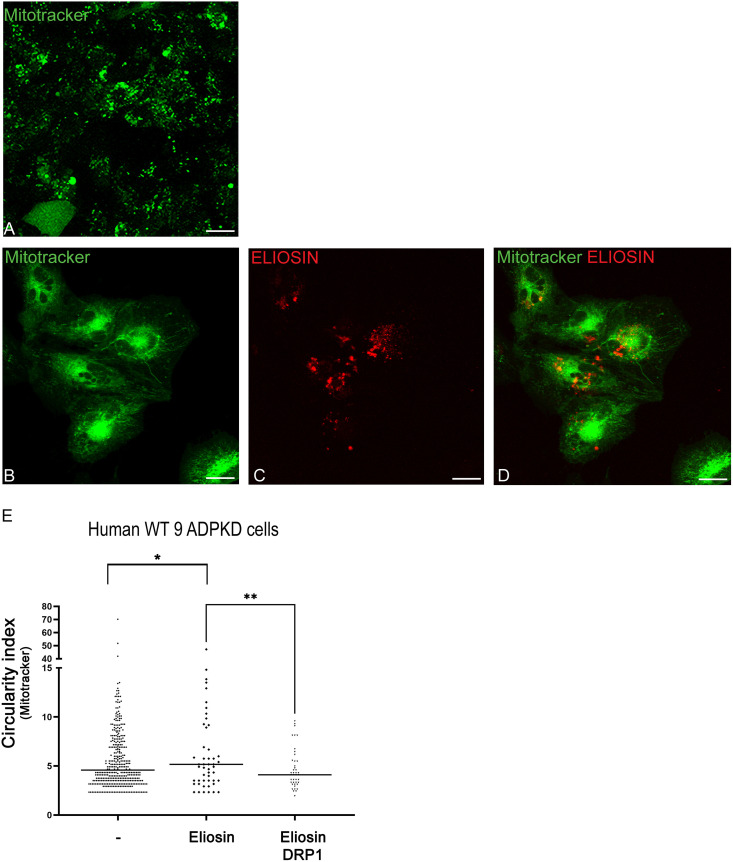
Eliosin can correct the fragmented mitochondria in ADPKD renal cells. A. Untransfected WT 9−7 cells labeled with Mitotracker green show punctate mitochondria (Top panel). Bar = 10 microns. B-D. WT 9−7 cells labeled with Mitotracker green (B) and transfected with mCherry-Eliosin (red) (C) result in filamentous mitochondria. The merged image (D) shows cells with extended reticular mitochondria. Bar = 10 microns. E. Graph of mitochondria circularity measurements in WT 9−7 untransfected cells labeled with Mitotracker versus WT 9−7 cells transfected with Eliosin or transfected with Eliosin and DRP1 and labeled with Mitotracker. * p < 0.001, ** p < 0.0002.

To interrogate Eliosin’s implication in the process of cyst associated mitochondrial defect, immortalized renal cyst cell line WT 9−7 that have fragmented mitochondria [45] served as a system model to evaluate mitochondrial functional defects with Mitotracker. In non-transfected WT 9−7 cells, the mitochondria are fragmented and scattered throughout the cytoplasm (Fig 7A). Upon transfection of Eliosin, the WT 9−7 cells mitochondrial phenotype is strikingly improved with a typical filamentous morphology (Fig 7B). This change in single cell analysis correlated with expression of the mCherry Eliosin chimeric protein (Fig 7C) and Mitotracker co-staining (Fig 7D). To quantify the mitochondrial morphology and dynamics in presence or not of Eliosin in ADPKD cells, we measured mitochondria circularity (Fig 7E). The WT 9−7 cells transfected with Eliosin cells show a mean mitochondria elongation increased by 88% relative to untransfected cells (p < 0.01), suggesting that Eliosin can interfere with mitochondria fission. Addition of DRP1 to Eliosin in WT 9 cells by transfection abrogated the apparent repair process of mitochondria fragmentation. This data points to a functional role of Eliosin in the mitochondrial fission and potential fusion process.

## Discussion

Autosomal dominant polycystic kidney disease is caused by multiple kidney epithelial cell growth derangements resulting in cyst formation [46]. The more common causative genes Hm*PKD1* and Hm*PKD2* produce Polycystin1 and Polycystin2 respectively and both proteins form a heteromeric complex that fully assembles in monocilia [46]. Cilia signaling via this complex is activated by mechanical deformation or by exosomes resulting in calcium transients that activate store operated calcium signaling from the rough endoplasmic reticulum. Signaling failure from the cilia is associated with a variety of epithelia cell maturation defects leaving the cells in an immature state with increased proliferative potential [46]. The Hm*PKD1* gene locus analysis has proved to be quite challenging on several fronts due to the gene’s partial duplication and PC1’s complex biogenesis. In addition, Polycystin1 has widespan subcellular localization(s) with many proposed function(s) and associated signaling pathway(s). In this communication, we evaluated the transcripts that map to the Hm*PKD1* locus and report the finding of an alternative transcript in a non-duplicated region of the human *PKD1* locus. A highly homologous alternative transcript is detected in mice and is also expressed in kidneys, indicating that it could play a role in parallel or in combination with *PKD1* physiologically and/or in ADPKD progression. This alternative transcript encodes a newly identified protein we have named Eliosin, that is detected endogenously in human renal cells and co-localize with ERMCS/MAM. Most importantly, Eliosin can improve mitochondrial defects typically identified in ADPKD cells and may have a major impact on PKD disease progression.

Evaluation of the transcripts mapping to the Hm*PKD1* locus revealed to be more complex than originally believed. In humans, the current map identifies 40 transcripts, many of which are non-coding transcripts of uncertain function. When a heat map was constructed using publicly available database from RNAseq studies, it became apparent that the transcript encoding full-length Polycystin1 was one of the least expressed transcripts in the locus. The transcript that is the subject of this communication has the greatest level of expression compared to the other human transcripts. Interestingly, analysis of the *PKD1* locus uncovered three transcriptionally active domains with open chromatin status, one of which in the 3’ region corresponding to this alternative transcript prevalent in many cell types. The cDNA from this alternative transcript has several unique features. Firstly, the transcript start site initiates within intron 40 of the Hm*PKD1* gene. There is a splice junction between the 3’ end of exon 41 splice donor site and the intron 42 splice acceptor site leading to skipping exon 42 of the Hm*PKD1* gene. Subsequently, the RNA splicing process follows that of the Hm*PKD1* locus for exons 43–46. Notably, mutations affecting the sequence in this alternative transcript at the C-terminal end of *PKD1* locus have a poorer renal survival prognosis of ~20 years relative to mutations upstream of the alternative transcript in ADPKD patients [47], suggesting that this region encodes a product(s) of high pertinence. The importance of this alternative transcript was also validated in the mouse. Similar transcriptional organization with exon 42 skipping is observed in the murine transcript; however, the transcript expression level appears less pronounced in mice as compared to human expression levels. The increased alternative transcript expression in adult transgenic *Pkd1*^*WT*^ or ^*SB*^*Pkd1* when compared to its expression level in wild-type mice can be attributed to the increased copies of the *Pkd1* locus (10–15 copies). However, we cannot exclude that this upregulation results from development of the renal cystic disease, which is consistent with increased expression of *PKD1*/*Pkd1* gene in cystic epithelia of murine models and human ADPKD kidneys [48]. The evolutionary conservation of this alternative transcript between human and mice suggests similar genomic and genetic regulation with possible conserved functional properties.

We elucidated the structural and biochemical properties of the protein Eliosin encoded by the alternative transcript from experiments on the open reading frame, protein initiation site, proteomic and biochemical analysis. The lack of an identifiable Kozak sequence or a canonical ATG start site particularly as the transcript was classified as non-protein coding raised questions on the *PKD1*–208 translation. The results of moving the cDNA into the cTAP plasmid in three reading frames uncovered a 47-48kDa open reading frame of expected size for Eliosin from the alternative transcript. Proteomic studies identified amino acid sequences that match the predicted protein sequence (Fig 4C). Importantly, the translation start site was determined from analysis of protein expression following mutagenesis of potential non-canonical initiation codon by deletion or point mutation. We identified a leucine as the translation start site corresponding at the nucleotide position 111 in the 126 bases of exon 41. This initiation start site produces 5 amino acids at the N-terminal region that are unique to Eliosin and differs from PC1’s amino terminal sequence. The structure of the start sequence matches criteria for non-canonical AUG translation initiation codon, with a cognate start codon CUG/leucine [49,50]. Interestingly, proteins initiating with leucine often generate or regulate proteins with key cellular functions particularly in response to cues of ER or oxidative stress, or heat shock conditions as well as in disease states [50]. In fact, initiation from Leucine in stress conditions was shown for the upstream promoter of the c-*Myc* gene [51] that can cause PKD [52,53] and of the p38MAPK-activated protein kinase *MK2* gene is also implicated in PKD [54]. The subsequent amino acid sequence of Eliosin is identical and in-frame with the last 399 amino acids of the carboxyterminal of PC1 with a predominant match to the last 200 amino acids identified by BLAST search. The findings are consistent with the proteomic analysis and in good agreement with the 2D gel showing an apparent pI greater than 9.0. Hence, Eliosin protein sequence does not encompass the G protein-coupled receptor (GPCR) autoproteolytic cleavage site (GPS) nor the p100 but may undergo cleavage like PC1 carboxy-terminal tail (CTT) and produce p ~ 30/34 and p ~ 15/17 fragments. The p34 could be consistent with a second band observed from protein translation of the Eliosin cDNA. Alternatively, this second smaller band could be explained by a cryptic protein coding initiation site. Moreover, the 48kDa Eliosin protein’s existence was confirmed by the predicted electrophoretic and charge mobility and by endogenous expression in human HK2 cells. Collectively, such converging results provide solid evidence for this new protein, Eliosin. These findings open the way for possible intriguing mechanisms that may control Eliosin transcriptional, translational and post-translational regulation in parallel with that of *PKD1*/PC1 and remain to be explored.

Our studies designed to evaluate Eliosin’s subcellular distribution revealed punctate pattern of Eliosin with positive staining over seemingly the mitochondria and ER using the anti-human PC1 in HEK 293 cells. Similar mitochondria and RER pattern of Eliosin chimeric protein SNAP- or mCherry-tagged in HEK 293 cells significantly reinforced our sublocalization observations. Moreover, its co-localization with all three proteins located at mitochondria-ER contact site; IP3R, mitofusin-2 and DRP1 [20,51] strongly supports the conclusion that Eliosin is a component of mitochondria-ER contact sites. While Eliosin appears preponderantly in ERMCS/MAM, it does not exclude that Eliosin may be present in other subcellular compartments. Interestingly, the Eliosin localization to ERMCS/MAM correlates with the concept that protein initiating with leucine are frequently implicated in response to stress conditions and can provide significant clues on Eliosin functional role in relation to energy production and metabolic processes. In our co-localization studies with DRP1, we found two outcomes: 1) co-localization between DRP1 and Eliosin in ERMCS/MAM punctate structures or 2) Eliosin localization to mitochondria staining pattern and DRP1 diffusely located in the cytosol (S4 Fig). This finding indicates that Eliosin may compete with DRP1 for mitochondria outer membrane proteins that bind to DRP1 such as MiD51, MiD49, Mff and Fis1. These mitochondrial factors mediate DRP1 assembly on the mitochondria outer membrane and are the factors that are required for mitochondria fission [55]. Upon co-localization, Eliosin could recruit and even interact with DRP1 or possibly sequester DRP1 at the ERMCS/MAM from usual interactions. Hence, Eliosin may prevent interaction of DRP1 with mitochondria outer membrane and thereby shift the balance from membrane fission mediated by DRP1 to membrane fusion catalyzed by mitofusin [29].

The functional role of Eliosin with DRP1 in mitochondria membrane fusion/fission process was addressed in immortalized renal epithelial line, WT 9−7 cells which are heterozygous for a *PKD1* mutation and are derived from ADKPD cysts [45]. These ADPKD cells display fragmented mitochondria [45] as also observed in normal renal epithelial cells following acute kidney injury [47]. In contrast, normal renal epithelial cells mitochondria form extended filamentous reticulum *in vivo* [45,47,56]. Generally, mitochondria morphology ranges from small spheres or ovoids to filamentous arrays depending on the tissue and cell type [29,53–55]. Fragmented mitochondria visible by Mitotracker in these ADPKD cells, with presumed Myc upregulation [45], a master regulator of genes in mitochondria dynamics including DRP1 [57], become upon Eliosin transfection, filamentous resembling differentiated cell mitochondria. Moreover, circularity index analysis of mitochondria morphology also concurs to attribute a role of Eliosin in the mechanism of either promoting mitochondria [45] elongation or inhibiting mitochondria fission. These results demonstrate that Eliosin can partially rescue ADPKD fragmented mitochondria. It also suggests that Eliosin could improve cellular defects and ultimately modify disease progression. Such findings could also be the result of putative processing of Eliosin, since a low molecular weight fragment of PC1 C-terminal tail has been reported to localize to mitochondria and alter its physiology [18,58,59] through a yet undetermined molecular mechanism. Hence the discovery of Eliosin raises additional complexity and concerns on functions attributed to PC1 C-terminal proteolytic or other PKD1 derived gene products in mitochondria. Conversely, the mitochondria circularity of the ADPKD cells was exacerbated by co-transfection of Eliosin with DRP1 that promotes mitochondria fission, supporting DRP1 in opposing the molecular mechanism of Eliosin in the mitochondria. Moreover, these data support the hypothesis that Eliosin is sufficient, probably directly, to regulate and shift the dynamic between mitochondria fission and fusion by interfering with DRP1 activity or mitochondria membrane assembly [29].Terminally differentiated renal epithelial cells, mitochondria form an extended filamentous reticulum *in vivo* [56]. This cellular activity can correlate with cellular differentiation state whereas in undifferentiated stem cells, mitochondria are fragmented [60]. Interestingly, in the PKD murine *Ksp-Cre;Pkd1*^*flox/-*^ model, cystic cells have fragmented mitochondria and inhibition of DRP1 with Mdivi-1 not only reverses mitochondria fragmentation but also improves the cystic phenotype [61]. Since Eliosin can also correct mitochondria fragmentation this may account for the improved renal survival prognosis in ADPKD patients with a mutation upstream of Eliosin coding sequences [47].

Polycystin1 fragments, with mitochondria localization, are reported to alter mitochondria physiology [18,58]. More recent evidence identified a caspase dependent 15kDa cleaved Polycystin1 product that fragments mitochondria [59]. This work links Polycystin1 to oxidative stress induced mitochondrial fragmentation. Our preliminary studies however have not revealed a difference in mitochondria fatty acid substrate utilization associated with Eliosin expression but needs to be confirmed.

In summary, Eliosin is the product of a unique transcript from the 3’ end of the Hm*PKD1* and murine *Pkd1* locus. Discovery of the alternative Eliosin transcript and protein uncovered further intricacies of the *PKD1* locus. Our results also find that Eliosin plays a specific role in the mitochondria-ER attachment sites as a modifier of mitochondria dynamics with potential impact on PKD disease progression. Future studies on Eliosin will be important to identify interactions with other proteins, their regulatory factors and molecular function that could pave the way to therapeutic design of diseases with mitochondria pathology.

## Supporting information

S1 Fig Expression profile of all the *PKD1* transcripts across multiple tissues obtained from GTEx. X-axis shows the human body sites reposited in GTEx database. The expression matrix was normalized by column maximum and represented as hierarchically clustered heatmap where the transcript ids (ENSTs) and names were labeled in y-axis (on right) as annotated in Ensembl database (GRCh38.p12). The upper part of the heatmap are transcripts with higher expression represented also in Fig 1, and the lower part of the heatmap are transcripts with low expression. The dendrogram on left represents expression-based clustering.
(PDF)

S2 Fig Comparison of the human and mouse *PKD1* locus A. Human *PKD1* locus from 5’UTR to 3’UTR showing CpG density profile and histone modifications over the entire *PKD1* locus (Human Browser). The extended view of the stippled region is shown in Fig 3A. B. Mouse *Pkd1* locus from 5’UTR to 3’UTR showing candidate cis-regulatory elements cCREs with promoter-like and enhancer-like signatures and histone modifications over the entire *Pkd1* gene (Mouse browser). The extended view of the stippled region is shown in Fig 3A.
(PDF)

S3 Fig Scanning map of the mouse *Pkd1* transcript A. Mouse *Pkd1* transcriptional activity was analyzed with different sets of primers. Two pairs of primers in exon 46 (46’ and 46”) have a common forward primer but have different reverse primer. B. The entire locus was scanned using wild type mouse kidneys (C57BL6/J) cDNA at postnatal day 20 as a template and was monitored by qPCR for analysis of amplicons. C. The corresponding melting curve of PCR amplified products. The region 5’ from exon 1 to exon 40 resulted in a single peak and one product. From exon 41 to the 3’ end of the *Pkd1* transcript region, double peaks from the melting curve were clearly detected and appearance of extra amplicons. NTC, non-template control; + , template cDNA; M, 1Kb plus DNA ladder, molecular weight marker.
(PDF)

S4 Fig Cellular localization of Eliosin and DRP1 HEK 293 cells were transfected with mCherry Eliosin (red) and DRP1 green fluorescent protein (GFP). Note the diffuse cytosolic expression of DRP1 GFP versus the punctate stain of the mCherry Eliosin suggests that DRP1 can be displaced into the cytosol with Eliosin transfection. Bar = 10 µm.
(PDF)

S5 Fig Pairwise alignment of human and mouse alternative transcript A. Sequence alignment (NCBI BLAST) of the mouse kidney cDNA PCR product primers 23.01 (ex41–43^jct^) and 11.07 (ex45) indicated in Fig 3, sequenced by 11.07 primer (ex45) relative to the mouse wild-type *Pkd1* transcript (ensembl). B. Sequence alignment of the PCR product in A. relative to the human alternative *PKD1* transcript contig shown in Fig 2A. Color code for exons is shown below the alignment and same as in Fig 2.
(PDF)
