# Supplementary figures and images for: Eliosin-an alternative product from the HmPKD1 locus is a component of endoplasmic reticulum mitochondria membrane contact sites
Source: PLoS One. 2025 Oct 30;20(10):e0332969. doi: 10.1371/journal.pone.0332969 (PMC12574933; doi:10.1371/journal.pone.0332969)

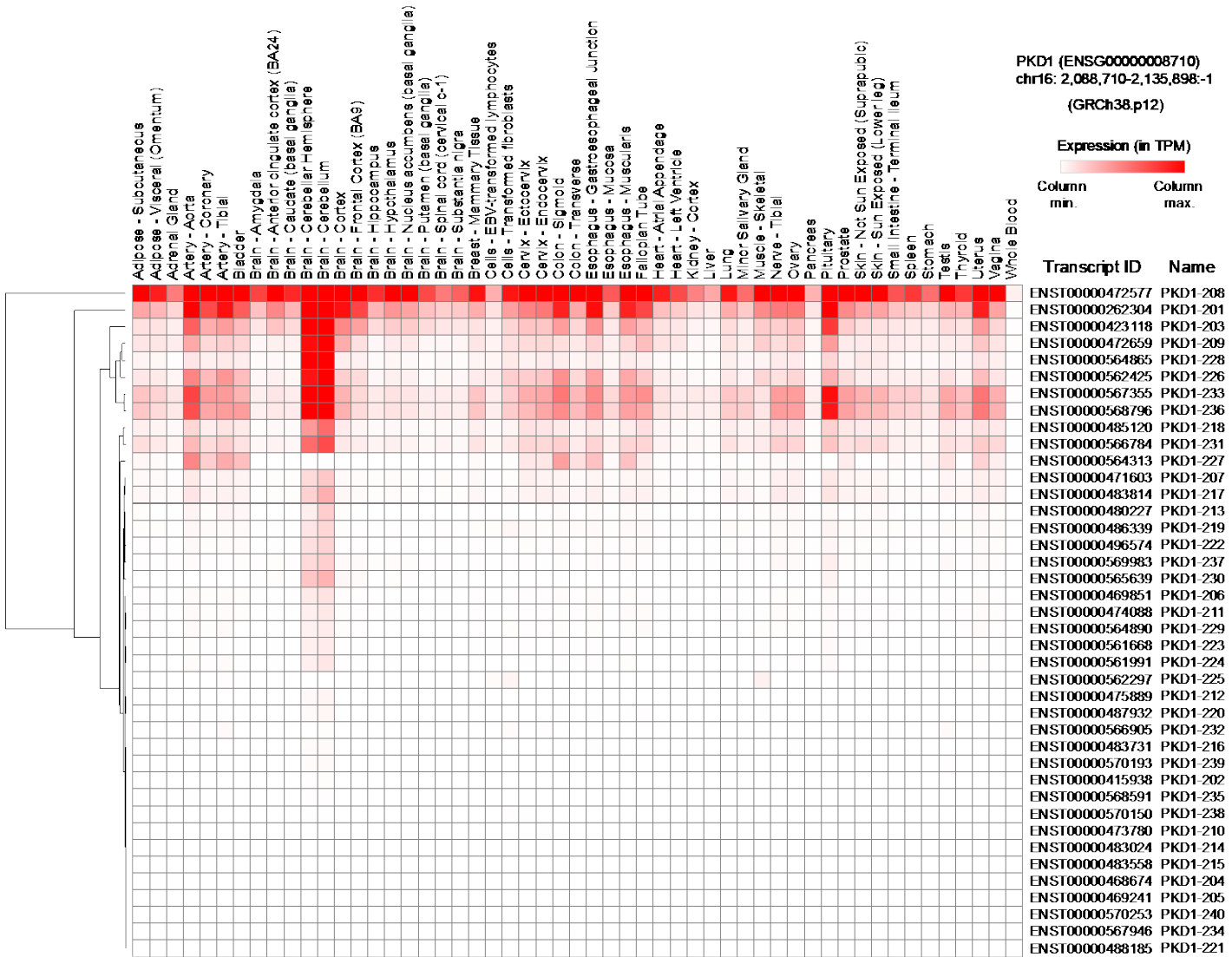

Supplement: S1 Fig — X-axis shows the human body sites reposited in GTEx database. The expression matrix was normalized by column maximum and represented as hierarchically clustered heatmap where the transcript ids (ENSTs) and names were labeled in y-axis (on right) as annotated in Ensembl database (GRCh38.p12). The upper part of the heatmap are transcripts with higher expression represented also in Fig 1, and the lower part of the heatmap are transcripts with low expression. The dendrogram on left represents expression-based clustering. (PDF) [file pone.0332969.s001.pdf]

A Human locus

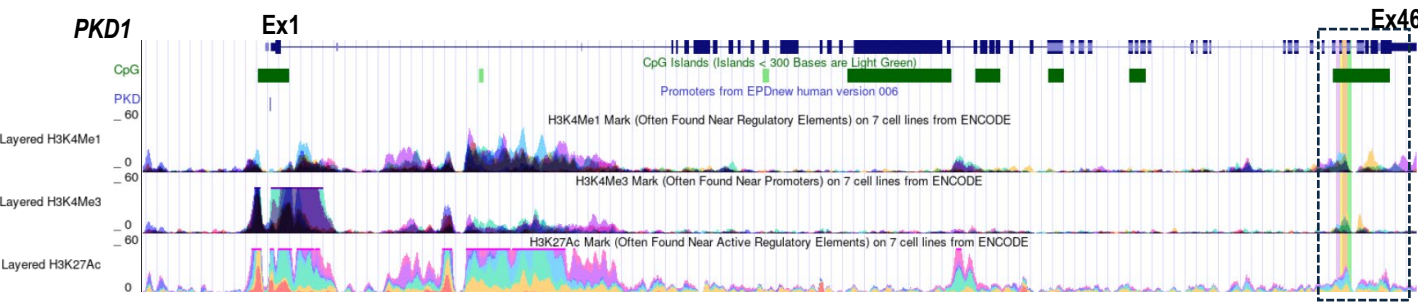

B Mouse locus

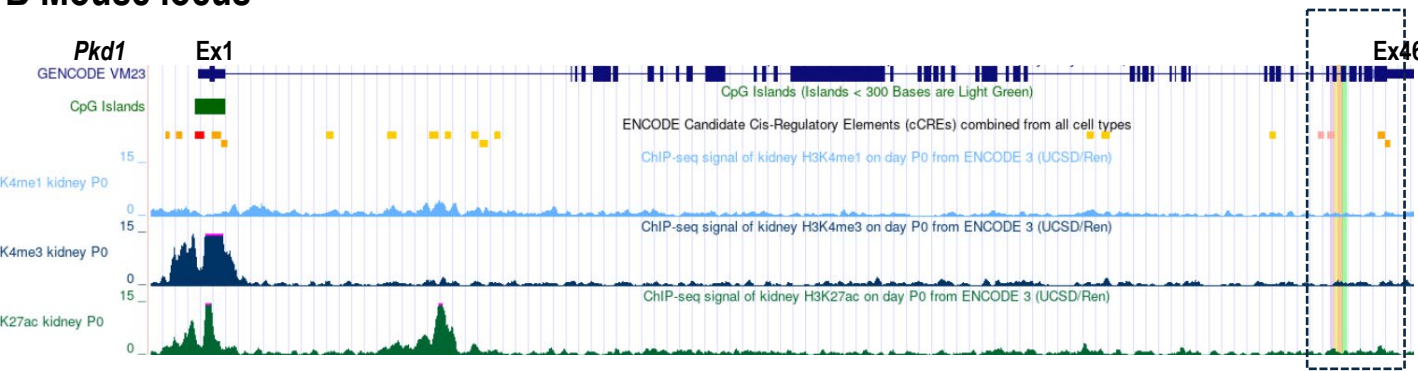

Supplement: S2 Fig — A. Human PKD1 locus from 5’UTR to 3’UTR showing CpG density profile and histone modifications over the entire PKD1 locus (Human Browser). The extended view of the stippled region is shown in Fig 3A. B. Mouse Pkd1 locus from 5’UTR to 3’UTR showing candidate cis-regulatory elements cCREs with promoter-like and enhancer-like signatures and histone modifications over the entire Pkd1 gene (Mouse browser). The extended view of the stippled region is shown in Fig 3A. (PDF) [file pone.0332969.s002.pdf]

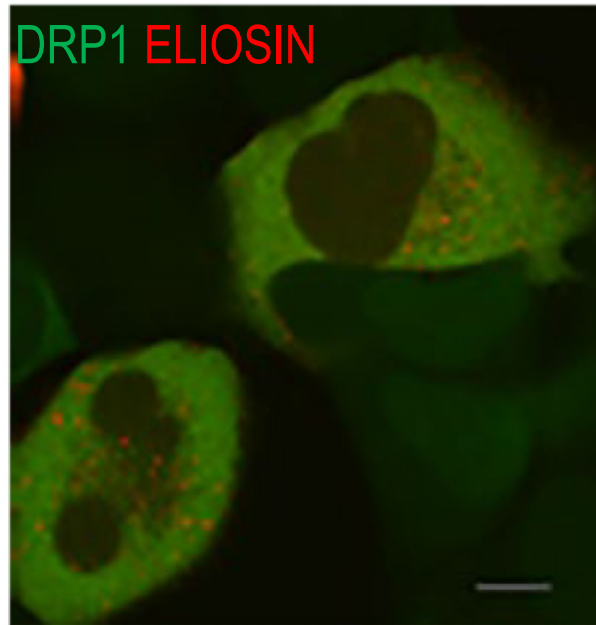

Supplement: S4 Fig — Note the diffuse cytosolic expression of DRP1 GFP versus the punctate stain of the mCherry Eliosin suggests that DRP1 can be displaced into the cytosol with Eliosin transfection. Bar = 10 µm. (PDF) [file pone.0332969.s004.pdf]

A Mouse vs mouse

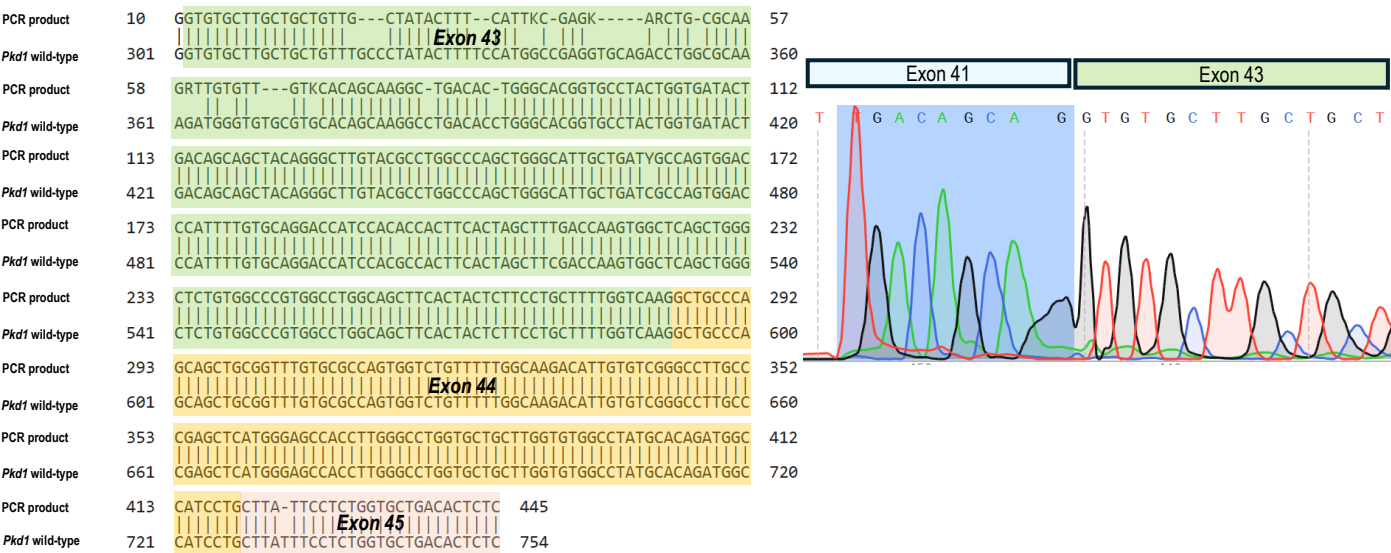

B Mouse vs human

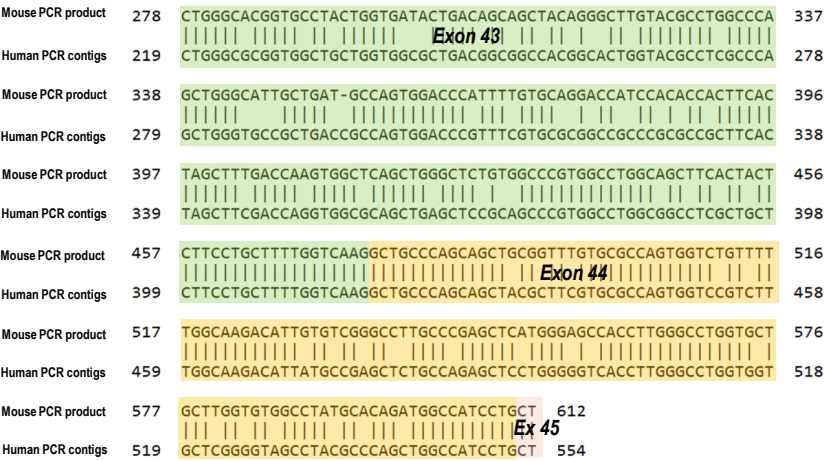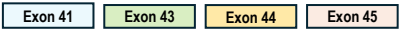

Supplement: S5 Fig — A. Sequence alignment (NCBI BLAST) of the mouse kidney cDNA PCR product primers 23.01 (ex41–43jct) and 11.07 (ex45) indicated in Fig 3, sequenced by 11.07 primer (ex45) relative to the mouse wild-type Pkd1 transcript (ensembl). B. Sequence alignment of the PCR product in A. relative to the human alternative PKD1 transcript contig shown in Fig 2A. Color code for exons is shown below the alignment and same as in Fig 2. (PDF) [file pone.0332969.s005.pdf]
