# Supplementary material for: Eliosin-an alternative product from the HmPKD1 locus is a component of endoplasmic reticulum mitochondria membrane contact sites
Source: PLoS One. 2025 Oct 30;20(10):e0332969. doi: 10.1371/journal.pone.0332969 (PMC12574933; doi:10.1371/journal.pone.0332969)

Supplemental Figure 3

A

Mouse *Pkd1*	Primer specific sequences
**Exon 1-2**	5' TCAATTGCTCCGGCCGCTG / 5' CCAGCGTCTGAAGTAGGTTGTGGG
**Exon 26-27**	5' GCTGGAGCCGAGGTTCAG / 5' TGTCCTCTCCATACAGCATGATG
**Exon 39-40**	5' CTGATGAGTTCTGGCCATGGATG / 5' CTGCCAGCCAATGCCATAGTCAC
**Exon 40-42**	5' GTGACTATGGCATTGGCTGGC / 5' ACTGAAGGCAGCCAGT
**Exon 41-45**	5' GCTACTGTGCAGTGTATGACA / 5' CCAGGGCACAGCACCAGGAA
**Exon 46'**	5' CACTTCTCCTTTATCCGTCCC / 5' CCAATTGCTGTCCAGCACCTG
**Exon 46"**	5' CACTTCTCCTTTATCCGTCCC / 5' TTCATCCCACAGTAGTCCTG

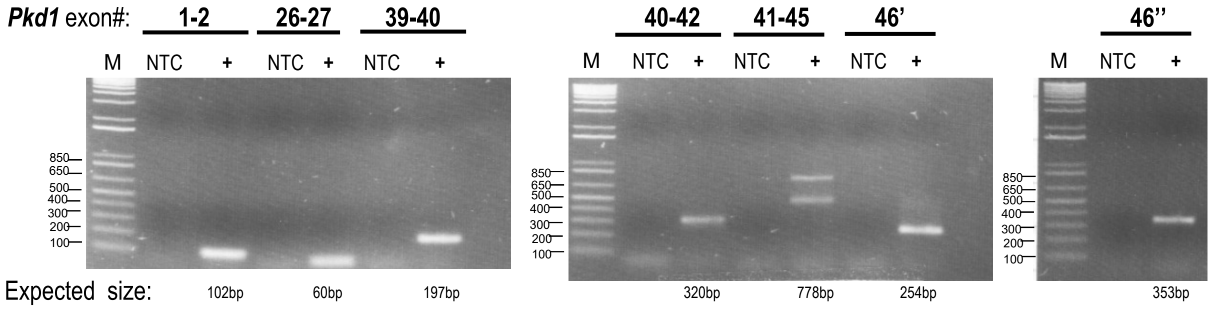

B

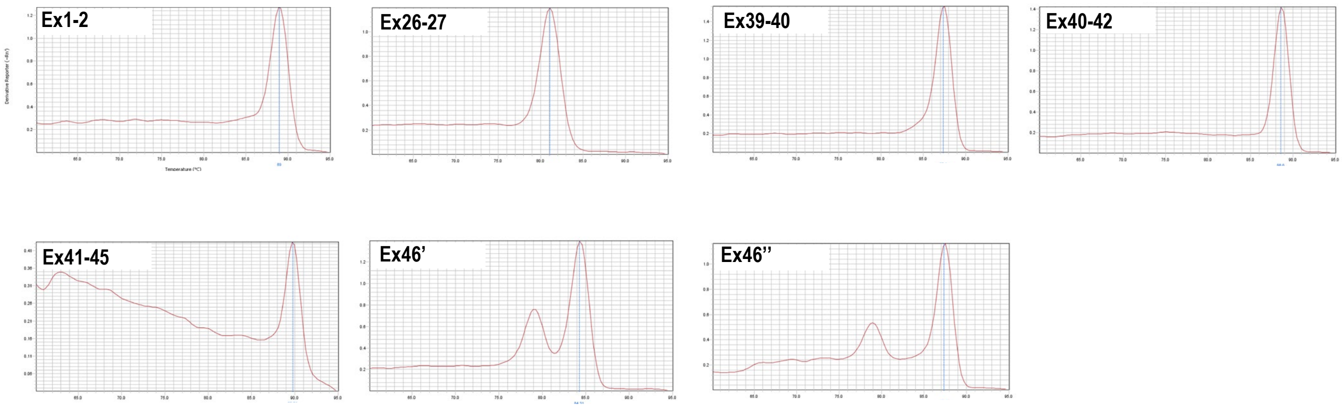

Supplement: S3 Fig — A. Mouse Pkd1 transcriptional activity was analyzed with different sets of primers. Two pairs of primers in exon 46 (46’ and 46”) have a common forward primer but have different reverse primer. B. The entire locus was scanned using wild type mouse kidneys (C57BL6/J) cDNA at postnatal day 20 as a template and was monitored by qPCR for analysis of amplicons. C. The corresponding melting curve of PCR amplified products. The region 5’ from exon 1 to exon 40 resulted in a single peak and one product. From exon 41 to the 3’ end of the Pkd1 transcript region, double peaks from the melting curve were clearly detected and appearance of extra amplicons. NTC, non-template control; + , template cDNA; M, 1Kb plus DNA ladder, molecular weight marker. (PDF) [file pone.0332969.s003.pdf]
